# Crowd Sensing-Enabling Security Service Recommendation for Social Fog Computing Systems

**DOI:** 10.3390/s17081744

**Published:** 2017-07-30

**Authors:** Jun Wu, Zhou Su, Shen Wang, Jianhua Li

**Affiliations:** 1 School of Electronic Information and Electrical Engineering, Shanghai Jiao Tong University, Shanghai 200240, China; junwuhn@sjtu.edu.cn (J.W.); lijh888@sjtu.edu.cn (J.L.); 2 School of Mechatronic Engineering and Automation, Shanghai University, Shanghai 200444, China; 3 School of Marxism, Zhejiang University of Science and Technology, Hangzhou 310023, China; shen.wang@zust.edu.cn

**Keywords:** fog computing, social networks, service discovery, service recommendation

## Abstract

Fog computing, shifting intelligence and resources from the remote cloud to edge networks, has the potential of providing low-latency for the communication from sensing data sources to users. For the objects from the Internet of Things (IoT) to the cloud, it is a new trend that the objects establish social-like relationships with each other, which efficiently brings the benefits of developed sociality to a complex environment. As fog service become more sophisticated, it will become more convenient for fog users to share their own services, resources, and data via social networks. Meanwhile, the efficient social organization can enable more flexible, secure, and collaborative networking. Aforementioned advantages make the social network a potential architecture for fog computing systems. In this paper, we design an architecture for social fog computing, in which the services of fog are provisioned based on “friend” relationships. To the best of our knowledge, this is the first attempt at an organized fog computing system-based social model. Meanwhile, social networking enhances the complexity and security risks of fog computing services, creating difficulties of security service recommendations in social fog computing. To address this, we propose a novel crowd sensing-enabling security service provisioning method to recommend security services accurately in social fog computing systems. Simulation results show the feasibilities and efficiency of the crowd sensing-enabling security service recommendation method for social fog computing systems.

## 1. Introduction

The concept of fog computing was presented to make the cloud computing framework better suited for data sources, such as sensors, in the Internet of Things (IoT) [1]. The core idea of fog computing is front-end intelligent, that is, using special equipment or network equipment to provide computing, memory, and network communications services between the cloud server and terminal equipment. Recently, fog computing has been regarded as a new networking and computing model for sensor networks, Internet of vehicles, smart grids, etc. Therefore, it brings great benefits by integrating fog computing and existing networks. Meanwhile, for the objects from the IoT to the cloud, it is a new trend that these objects establish social-like relationships with each other, with the benefits of efficiently bringing developed sociality to a complex environment. The integration can form social relationships with the benefits of network boundary extensions and network scalability. Thus, the social attributes and capabilities can be assigned to the nodes in communication networks, which makes them important parts of social networks. Recently, the social networking model has been introduced into advanced social networking, sensor networks, smart grids, the cloud, etc. The basic model combining social networks and the Internet of things was proposed in [2], in which related benefits were discussed deeply. Moreover, some important topics, such as context awareness, service architecture, and trustworthiness management, etc., are studied widely [3,4,5], especially for typical application systems (e.g., social body sensor networks). Additionally, a novel incentive scheme for a cyber-physical social system (CPSS) was proposed based on the reputation of social users [6]. In addition, University of Glasgow, UK, proposed a real application system of the social IoT in the smart city, in which the sensors can be integrated with the social network system (e.g., Twitter), thus the citizens can use and share the sensing data and improve the intelligence of the smart city [7]. Moreover, a novel framework was proposed to deliver content in vehicular social networks with D2D communication [8]. At the same time, the social cloud is also a new resource and service sharing framework utilizing relationships established between members of a social network [9,10]. In the work of [11], a social virtual object architecture was proposed for the edge cloud. However, this work focused on the social edge cloud but not real social fog. Based on aforementioned analysis, although there are some existing social architectures for various networks, cloud and edge cloud, social fog is still an open issue. In fact there are several drawbacks in these existing systems. Firstly, in most social cloud computing systems, the social relationships are established for the concentrated cloud center, which cannot provide adequately flexible, secure, and collaborative services for the network edge. Secondly, in the existing social sensor networks, the social networking model is introduced among the sensor nodes. However, because of constrained computing and storage resources of the sensors nodes, the efficiency of the social organization is limited. Thirdly, in traditional fog computing systems, the social relationship for the fog nodes has not been considered, thus the efficient and flexible service needs to be improved.

By introducing the attributions and ideas of social networking into fog computing, the nodes in existing fog computing systems can establish social relationships autonomously. Furthermore, the nodes in existing fog computing systems can act as the social network nodes, thus, a bridge can be constructed between the network edge resources and social users. At the same time, social networking advancing fog computing systems get a lot of new benefits, but the features of complexity, openness and dynamics of social networks enhance the complexity and vulnerability [12,13]. There exist various kinds of services in fog computing systems and social networks, which make the service environments more complex. When the users face different kinds of services in social networking advanced fog computing systems, it is a very important issue to recommend security services to the users [14,15,16]. Meanwhile, some service recommendation architectures are proposed for traditional networks. For example, the work in [17] proposed a novel content-based service recommendation mechanism, which considers simultaneously semantic content data and rating data of web services. Moreover, a reputation measurement method for web service recommendations was proposed in [18], which can enhance obviously the success ratio of service recommendation. In addition, the service recommendation approach proposed in [19] employs the features of Quality of Services (QoS), in which the recommendation visualization technique is used. Thus, it is visual for the users how a recommendation is grouped with other services choices. Although there are a lot of existing service recommendation schemes, these existing works cannot satisfy the requirements of security service recommendations in social networking advanced fog computing systems with randomness, complexity, and time-dependence features. Therefore, these existing service recommendation schemes cannot be used directly in social fog computing.

The state of the art of the idea combining fog computing with social networking includes the following points. Firstly, social fog computing systems can establish social relationships in an autonomous way with the benefits of extending the fog boundaries and enhance the scalability of fog computing systems. Secondly, in social fog computing systems, the fog nodes can obtain the social attributes and capabilities and become the key component of social networks. Thirdly, in social fog computing systems, the fog node establishes a bridge between the social users and the service computing systems. Moreover, the contributions of the paper include following points:

- We design an advanced social networking architecture for fog computing called social fog computing to control and organize fog computing system efficiently as well as securely;
- We propose a novel crowd sensing-enabling security service recommendation method for a social fog computing architecture. The computation model and parameters of security service discovery as recommendation are designed for social fog computing systems.

The rest of this paper is organized as follows: Section 2 presents the preliminaries of fog computing, as well as the technologies used in security service recommendations. In Section 3, the details of social fog are presented, including its motivation, innovations, and architecture. Section 4 shows the details of the proposed security service recommendation mechanism for social fog. Moreover, Section 5 evaluates the performance of the proposed security service recommendation mechanism. Section 6 concludes this paper.

## 2. Preliminaries

### 2.1. Fog Computing

Fog, as a localized cloud, provides networking, computation, and storage services between the end nodes, which includes a fog micro data center (MDC) and a fog smart gateway (FSG) as an additional security layer and data filtration [1]. Fog computing can be widely used in shopping centers, hotels, schools, hospitals and other places which are crowded and have specific goals, and it can also be applied to the systems of high delay requirements, such as grid sensing control closed-loop control system, vehicle infrastructure cooperative systems and so on. In addition, the fog computing has a large prospects in expanding coverage area of cloud computing, such as the ability to provide high availability of data and computing services in mountainous areas, buses, high-speed rail, airplanes and other areas. Fog computing gradually attracts more and more attention from researchers, academics begin to study fog computing security, the fog computing programming framework, and other specific key technologies.

### 2.2. Chance Discovery Theory

As a method beyond data mining, the purpose of chance discovery is to understand the meaning of rare chances (i.e., situations or events with important impact on decision-making) and support the optimal decision-making to prevent the system from various risks, where a chance is a situation or an event [20]. As one of the typical implementation methods of chance discovery, KeyGraph algorithm, proposed by Yukio Ohsawa et al., can extract the important points of the original data and map them as an intuitionistic graph [21,22]. Thus, the analysis for data can transfer into modelling and studying the graph. Recently, chance discovery has been used widely to perform social network analysis, natural disaster simulating, keyword extraction, earthquake prediction, etc. Chance discovery can also be used to perform the security service discovery in social fog computing systems.

### 2.3. Crowd Sensing

With the rapid developments of networking technologies, all kinds of sensors can act as the computing devices which cooperates each other. Crowd sensing computing is a novel technology in which a lot of users perform the cooperative computations based on networking nodes consciously or unconsciously [23,24]. Then the complex task can be implemented based on collective intelligence. Related concepts include crowd computing, participatory sensing, social sensing, crowd sourcing, etc. Nowadays, crowd sensing is a novel kind of distributed mobile computing model, which has attracted a lot of attentions [25]. Crowd sensing is introduced in this paper to realize the security service recommendation.

## 3. Proposed Social Fog Computing Systems

### 3.1. Design Motivation of Social Fog Systems

The basic idea is the definition of a social networking advanced fog computing system, named social fog. Based on the existing studies on social networking, especially about the features and architecture, we argue that the social networking model is useful for fog computing systems because the advantages meet fog computing systems’ requirements very well.

Firstly, the social networking approach can help fog computing systems provide better resource management. As a matter of fact, with the rapid developments of fog computing technologies, an explosive growth of complex relations and connections in fog computing systems will be included inter and intra fogs. Thus, the social attributions become more and more important in fog computing systems. Social networking can address this problem effectively through organizing efficiently the resources in the fog computing systems.

Secondly, the social networking approaches that can help fog computing systems support the data are classified and forwarded by content-aware traffic control in fog computing systems. Based on the existing studies on social networks and fog computing systems, fog devices expect to focus on the data content they want to access regardless of where that data content is stored. In addition, provisional users in fog computing systems do not need to retrieve data upon reconnection to the fog computing systems instead of obtaining the history data.

Thirdly, social networking self-contained security services can help fog computing systems simplify the processing of security. Fog computing systems have an important security requirement to ensure data confidentiality, integrity, and authenticity. Especially, it is a very important topic for users to discover the security services. The efficient organization model for social networks benefits the security service discovery for fog computing.

### 3.2. Innovations from Fog to Social Fog

Being similar to social network services (SNS) for human beings, the novel model establishes the social relationship model among the resources of fog computing systems. The innovations and benefits from fog to social fog are shown as follows. Firstly, the fog computing systems with the social organization effectively improve the performance of object and service discovery by modelling the social relations in human social networks. Secondly, if the social network models are introduced into the fog computing systems, the nodes in the fog computing systems can be managed efficiently to deal with complex communication and computation environments. Thirdly, the “friends” and trustworthiness relations can be established among the fog computing nodes. The proposed social fog is expected to guarantee a higher scalability, thus, an efficient interoperation can be provided among the objects involved in the fog computing systems.

### 3.3. Social Fog Architecture

**Figure 1:**
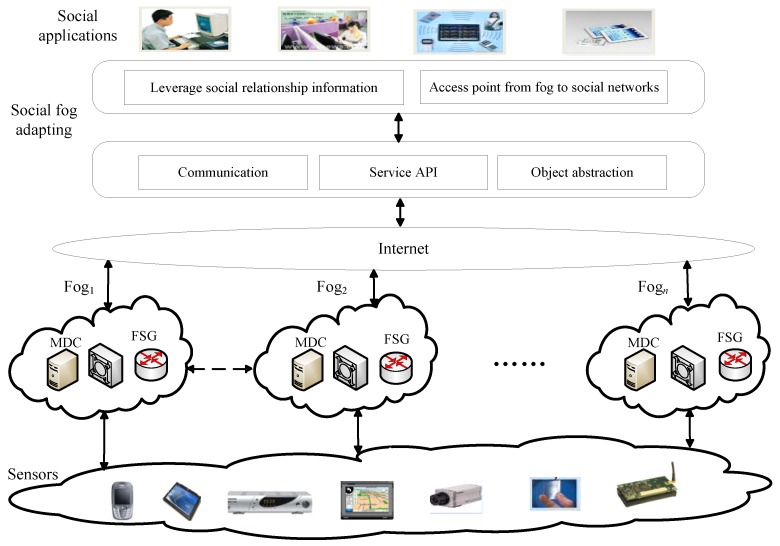
Service scenario of social fog.

Social networking advanced fog computing systems include a number of fogs between the sensing layer and social application layer. As is shown in Figure 1, it contains four layers; respectively are the social application layer, service layer, fog layer, and sensor layer. 

For the limited resource sensor nodes, it only needs to send sensing data to the fog as a source node, or receive the data as a destination node. For the local fog, it can only focus on local sensors and does not care about other fogs’ and sensors’ states. Moreover, after the fog receives the sensing data sent from a sensor, it only needs to process the data content and send them to the social fog adapting layer. The social fog adapting layer provides access to the necessary aspects of users’ social networks. Sociotechnical adapters can leverage any source of social relationship information, such as an existing social network platform. The social fog adapting layer can be used to access social information in real time or it can statically or periodically retrieve users' information. In this model, state data is collected by fogs and sent to the service layer. The service layer can provide the security service discovery capabilities for social applications.

## 4. Proposed Security Service Recommendation Mechanism

### 4.1. Architecture of the Security Service Recommendation of Social Fog

**Figure 2:**
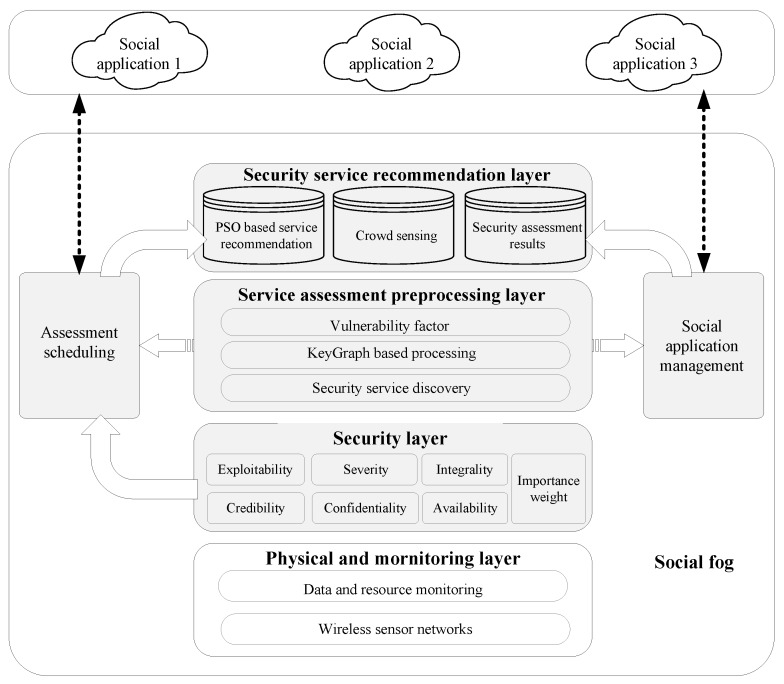
Node structure of social fog.

With the goal of realizing a security service recommendation for a social fog system, we propose a KeyGraph and crowd sensing-enabling efficient service provisioning scheme for fog nodes, which is shown in Figure 2. 

The proposed hierarchical architecture is composed of a physical and monitoring layer, security layer, service assessment preprocessing layer, and transportation layer over social networks. In the security layer, security factors, including confidentiality, integrity, availability, exploitability, credibility, and severity are used as the security factors of services for assessment. Vulnerability factor extraction, KeyGraph-based preprocessing, and security service discovery are implemented in the service assessment preprocessing layer. Particle swarm optimization (PSO)-based crowd sensing-enabling security service recommendation can be performed in the security service recommendation layer. The users of social fog are enabled to use the security services recommended by the underlying fog nodes to their changing and dynamic requirements. The security service recommendation architecture of social fog is enriched with several extra functional operations. Based on the service policy distribution from social applications, preprocessing orchestration should be implementation to support the security service assessment and discovery. The assessment scheduling module processes the operations sequence of security service assessment, discovery and recommendation. In addition, the social application management module performs the interoperations between the social applications and fog nodes. 

There are two phases in the proposed security service recommendation method. The first phase is to assess and discover a security service. The second phase is to search for the best global security service in the service swarm based on crowd sensing.

### 4.2. Security Services Assessment

Due to the randomness, as well as the openness, of the social environment and the virtualization of fog computing, the security assessment for the services is very complex in social advanced fog computing systems. At the same time, the security assessment scheme must satisfy the low-complexity requirements and deal with virtualization and dynamic environments. In this section, we propose a security service assessment scheme regarding the aforementioned factors.

Assume a service in the social networking advanced fog computing system is denoted as *fs*. The service set is denoted as *FS*, where fs∈FS. To assess the security of a service in social networking advanced fog computing system, confidentiality, integrality, and availability (CIA) are used as the basic assessment dimensions. Moreover, to make the CIA assessment dimensions uniform, a vulnerability factor is introduced into the assessment model. Here *vul* is used to denote service vulnerability and the set of vulnerability factors is denoted as *VUL*. The vulnerability factor of fs is denoted as *VUL*(*fs*). The vulnerability assessment and quantization methodology proposed by our previous work in [26] can be used to evaluate the vulnerabilities in dynamic and complex systems, and is based on an optimized attack graph and Analytic Hierarchy Process (AHP). Social fog computing is also a dynamic and complex computing and networking system, so the vulnerability assessment and quantization methodology in [26] is introduced. Moreover, assume *EX*(*vul*), *CR*(*vul*), and *SE*(*vul*) denote exploitability, credibility, and severity of *vul*, respectively. Then, *InSec*(*vul*) is used to denote the insecurity factor caused by *vul*, which can be computed by:

(1) InSec(vul)=EX(vul)×CR(vul)×SE(vul)

which is under the constraint of the value range (0,1]. In the security services assessment, the weighted insecurity factor of service is used to perform the assessment. The value of this factor means the insecurity level of the service. In other words, services with high security levels have low values for the insecurity factor. Thus, the most secure services can be found based on this factor.

Assume that *IISec* (*VUL, fs*) is the immediate insecurity on *fs* caused by *VUL*(*fs*), which can be calculated as:

(2) $$IISec(VUL,fs)=\{\begin{array}{c} 0,\;VUL(fs)=\emptyset \\ 1-\prod_{vul\in VUL(fs)}(1-InSec(vul)),\;VUL(fs)\neq \emptyset \end{array}$$

which is also under the constraint of value range (0, 1].

Next, in the security service assessment phase, assume that importance factor can describe the importance of service *fs*, which is denoted as *Imp*(*fs*). Moreover, the weighted insecurity of service *fs* can be denoted as (*Imp*(*fs*) × *IISec*(*VUL, fs*)). The weighted insecurity factor of service set *FS* can be computed as follows:

(3) $$InSec(FS)=\sum_{fs\in FS}\left(Imp(fs)\times IISec(VUL,fs)\right)$$

In addition, assume *UniInSec*(*FS*) is used to denote the uniformization of *InSec*(*FS*), which can be calculated by:

(4) $$UniInSec(FS)=\frac{InSec(FS)}{\sum_{fs\in FS}Imp(fs)}$$

Moreover, because the social networking advanced fog computing system is complex and dynamic, it is necessary to analyze transient state of the security service. To provide the analysis for the transient state of the security service, a finite homogeneous continuous-time Markov chain is used to establish the description model. Assume that {V(t),t≥0} denotes the finite homogeneous continuous-time Markov chain. Moreover, assume SP={1,2,…,n}=A∪B is the state space, where the absorbing state set is denoted as *A* and non-absorbent set is denoted as *B*. Assume *CH_i_*(*t*) denotes the transition probability matrix of the finite homogeneous continuous-time Markov chain. Let *CH_j_*(*t*) = *CH*(*SP*(*t*) *= j*), where *CH* is the transition probability matrix of the finite homogeneous continuous-time Markov chain, and *SP* is the state space. The transition probability matrix of the finite homogeneous continuous time Markov chain in *j*-th time slot is the transition probability matrix of the corresponding state space. Assume that infinitesimal generated matrix is denoted as *GM* = [*g_ij_*], then:

(5) $$\frac{dCH(t)}{dt}=CH(t)\cdot GM$$

where *CH*(0) is known. Moreover, within time [0, t], assume *Res*(*t*) is the reserve of state *j*. Here *Res*(*t*) can be obtained by:

(6) $$Res(t)=\int_{0}^{t}CH(\tau )d\tau$$

Then, the following formula can be obtained.

(7) $$\frac{dRes(t)}{dt}=Res(t)\cdot GM+CH(0),\;Res(0)=0$$

According to the transition in time slot T, GMT, which is the submatrix of GM, can be constructed. Then *GM_T_* can take the place of *GM* in Equation (7). $Res_{T}$ is the reserve of state *j* in time slot *T*. Next, assume $Res_{T}(\infty )$ can be obtained by:

(8) $$Res_{T}(\infty )=\underset{t\to \infty }{\lim}Res_{T}(t)$$

Assume *T_ab_* denotes the time when the system enters the absorbing state totally, which can be computed by:

(9) $$T_{ab}=\sum_{j\in T}Res_{j}(\infty )$$

### 4.3. Security Service Discovery Scheme

Security discovery should be performed before the security service recommendation. In this section, we use a KeyGraph-based chance discovery theory to design the security service discovery scheme.

#### 4.3.1. KeyGraph Establishment

In chance discovery theory there are several ways to perform the discovery. KeyGraph is a typical method in which the key points of the data are extracted. More importantly, the relations among important data can be mapped to an intuitionistic graph, thus, the computations for the relations of the important data can be translated into the analysis of the graph. In KeyGraph, the relations among the data correspond with the lines. Moreover, the tightness among the data can be quantified. Based on the advantages of KeyGraph, we use KeyGraph to design the security service discovery scheme. Firstly, the services in the service set *FS* are denoted as *fs*_1_, *fs*_2_, …,*fs_j_*, …, *fs_m_*. To map the relations of the services, each service *fs_j_* is regarded as a KeyGraph, which is denoted as KG*_j_*. To consider continuity of the time, assume that a service time includes *m* time slots, the service process during time *t_j_* to *t_j_*_+1_ is described based on the graph KG*_j_* presents. In KG*_j_*, an interoperability relation between two services is mapped to an edge, and a service of the social networking advanced fog computing system is mapped to a vertex of KG*_j_*. For each edge of KG*_j_*, the edge of the graph has the attributions in terms of the weight value and direction, where the number of the frequencies of the interoperability is mapped to the direction and the direction is from sender to receiver. The principle of the security service discovery follows.

#### 4.3.2. KeyGraph Connection Value

Assume the vertices whose frequency of out-edge beyond the threshold as the vertexes in KG*. Set *AC_i_* and *AC_j_* denote two vertices in KG*. *Association* (*AC_i_*, *AC_j_*) denotes the association between *AC_i_* and *AC_j_*, which can be computed by:

(10) $$Association(AC_{i},AC_{j})=\sum_{fs_{m}\in D}{\left|AC_{i},AC_{j}\right|}_{KG_{m}}$$

where $\left|AC_{i},AC_{j}\right|$ denotes the times of the directed line from *AC_i_* to *AC_j_*, which can be mapped to the service *fs_m_*. Based on the association value, the assessment for the tightness between *AC_i_* and *AC_j_* can be performed. In KeyGraph KG*, according to the association value between the pairs of vertices, they are sorted and identified. In other words, the tightness and relation of a pair of vertices can be assessed. A connected sub-graph called a cluster is used to denote a full procedure of security service discovery.

#### 4.3.3. Tightness Calculation

In KG*, it is very important to identify which nodes are security nodes. As a matter of fact, the vertices which are connected directly to the high-frequency cluster can be regarded as security nodes. *Tightness* (*X*) denotes the tightness of node *X*, and is computed as follows:

(11) $$Tightness(X)=1-\prod_{y\subset KG*}\left(1-Gra(X,y)/InteroP(y)\right)$$

where *y* is a cluster in the KeyGraph. In addition, *Gra*(*X*, *y*) is used to denote the nodes which connect the services with high frequency. *InteroP*(*y*) denotes the interoperation degree of node *X* in cluster *y*. Moreover, $H_{{\mathrm{KG}}_{m}}$ denotes the neighbor relationship of node *X* in cluster *y*. The layer value of vertex *X_y_* is denoted as *Lev* (*X_y_*).

(12) $$Gra(X,y)=\underset{fs_{m}\in U}{\sum }H_{G_{m}}(X,y)$$

(13) $$InteroP(y)=\underset{fs_{m}\in U}{\sum }\underset{X\in {\mathrm{KG}}_{m}}{\sum }H_{{\mathrm{KG}}_{m}}(X,y)$$

(14) $$H_{{\mathrm{KG}}_{m}}(X,y)={\left|XX_{y}\right|}_{{\mathrm{KG}}_{m}}\times Lev{\left(X_{y}\right)}_{X_{y}\in y}$$

Based on the above processing, the key values can be computed for all the vertices in KG*_m_*. Moreover, the nodes with higher security level will be added if they are not present in the KeyGraph. Most importantly, the infrequent and security service nodes with importance can be found, which is regarded as a candidate chance.

#### 4.3.4. Security Service Discovery

In KG*, service *X* connected with the key connections around it can be denoted as *H*(*X*, KG*). If the values of the service nodes are beyond a reasonable threshold, which are a set satisfying the security requirements of the applications, the service can be discovered as a security service.

### 4.4. Security Service Recommendation Based on Particle Swarm Optimization (PSO)

In this section, the security service recommendation mechanism is proposed for social fog computing. Particle swarm optimization (PSO) is a new bionic evolutionary algorithm for crowd sensing, which is inspired by the movement of birds looking for habitat [27]. PSO is introduced as a basis of the proposed crowding sensing based security service recommendation. In PSO, each individual can be regarded as a particle with no weight and volume. Although the moving track of a single particle seems chaotic, a particle can dynamically adjust the behaviors according to its own and its companions’ experiences until the swarm enters a better area of the environment with high fitness.

Assume that a particle swarm with *l* particles finds the optimal results in an *h*-dimension solution space. The PSO algorithm-based crowd sensing is as follows:

**Step 1:** Initialization: Set the learning factor *ler*_1_, *ler*_2_, and maximal evolution algebra *al_max_*, when evolution algebra *al* = 1. Assume that *m* service particles are generated randomly in space *R*, which are denoted as *p*_1_, *p*_2_, …, *p_m_*, and the service swarm matrix *sw*(*t*). Next, the displacement variations are generated randomly for each service particle, which are denoted as *c*_1_, *c*_2_, …, *c_m_* forming a displacement variation matrix *C*(*t*).

**Step 2:** The service swarm is evaluated, and the adaptive value *ADA*(*P_i_*) is computed.

**Step 3:** The adaptive value *ADA*(*P_i_*) of the current service particle is compared with its history optimization value *HisBe;* if *ADA*(*P_i_*) is better than *HisBe*, *HisBe* is set as the current value of *ADA*(*P_i_*), and the location of *HisBe* is set as the current location.

**Step 4:** The current adaptive value *ADA*(*P_i_*) is compared with the optimal value of the service swarm, which is denoted as *SwaBe*. If *ADA*(*P_i_*) is better than *SwaBe*, *SwaBe* is set as the current value of *ADA*(*P_i_*), where the order number of *SwaBe* is the order number of the current service particle.

**Step 5:** The new service swarm, denoted as *P*(*t* + 1), can be generated based on the updates of the velocity and location of the service particle. The location matrix of the *i*-th service particle is denoted as *Li* = (*l_i_*_1_, *l_i_*_2_, …, *l_id_*). Assume that the best location searched by the *i*-th service particle is *LOC_i_* = (*trac_i_*_1_, *trac_i_*_2_, …, tra*c_id_*), which is the location that the *i*-th service particle passed with the best adaptive value. *LOC_g_* = (*trac_g_*_1_, *trac_g_*_2_, …, *trac_gd_*) is used to denote the best location where all the service particles passed so far. The best adaptive value can be computed based on the objective function of the object problem. In the *t*-th step of the computation, assume the security assessment factor and service track of the *i*-th service particle in the *j*-dimensionality space are *loc_ij_*(*t*) and *v_ij_*(*t*), respectively:

(15) $$v_{ij}(t+1)=\partial v_{ij}(t)+a_{1}\left(loc_{ij}(t)-l_{ij}(t)\right)+a_{2}\left(loc_{gj}(t)-l_{ij}(t)\right)$$

(16) $$l_{ij}(t+1)=l_{ij}(t)+v_{ij}(t+1)$$

where *∂* is the inertial factor, and *a*_1_, *a*_2_ is the variation coefficients of the security assessment factor. Assume the *a*_1_ is an adjustment factor used to adapt the security assessment factor of the service particle based on its own optimal solution. Additionally, let *a*_2_ be an adjustment factor used to adjust the security assessment factor of the service particle adapting to the global optimal solution.

The current adaptive value *ADA*(*P_i_*) is compared with the optimal value of the service swarm *SwaBe*. If *ADA*(*P_i_*) is better than *SwaBe*, *SwaBe* is set as the current value of *ADA*(*P_i_*), where the order number of *SwaBe* is the order number of current service particle.

**Step 6:** The evaluation value is checked to judge whether it achieves a given accuracy. If the evaluation value achieves given accuracy, the circulation is finished. Otherwise, set *t* = *t* + 1 and jump to **Step 2**.

Next, the security service recommendation method is proposed. Assume that *N* is the matrix for predicting *n* historical security service samples and *M* is the prediction matrix of the *j*-th security service of the *N* by using *e* predication methods. Then *M* and *N* are standardized, and assume that *EM* and *EN* are the standardized matrixes of *M* and *N*, which are computed by:

(17) $$EM_{i}=\frac{N_{i}-\bar{N}}{std_{N}}$$

where $\bar{N}$ and *std_N_* are the mean value and standard deviation of *N*, respectively.

(18) $$EN_{ji}=\frac{M_{ji}-{\bar{M}}_{i}}{std_{i}}\;(j=1,2,\ldots ,n,\;i=1,2,\ldots ,e)$$

where ${\bar{M}}_{i}$ and *std_i_* are the mean value and standard deviation of the *i*-th independent variable, respectively.

Assume that $SR={\left[sr_{1},sr_{2},\ldots ,sr_{n}\right]}^{T}$ is the weight matrix of *n* service samples. Based on the weight matrix, the new matrices *M^n^* and *N^n^* are introduced. The construction method is as follows: Assume that the *p*-th sample occurs *β_f_* times in the original data matrices *M^n^* and *N^n^*. In fact, when the data volume increases, the dimensionality number and the computation complexity of the data are very high. To decrease the computation complexity, we introduce the new matrices *M^nn^* and *N^nn^*, which can be computed as follows:

(19) $$M^{nn}=diag\left(\sqrt{p}\right)M$$

(20) $$N^{nn}=diag\left(\sqrt{p}\right)N$$

where $diag\left(\sqrt{p}\right)$ denotes the diagonal matrix composed by the service elements which are the square roots of the sample weight matrix *p* = [*p*^1^, *p*^2^, …, *p^n^*]*^T^*. Partial least-squares (PLS) regression is a multivariate analysis method, which was proposed by Wold and lbano for some import regression problems, such as multicollinearity. PLS regression performs the integrations and selections, then extracts the aggregative variable with the best explanations for the systems. At the same time, PLS regression can delete the multicollinearity information and the information without explanation meanings, thus, it can resolve the problem of multicollinearity among the variables. Therefore, the model with good imitative effect, robustness, and prediction capabilities can be obtained. PLS regression can be used to analyze the mass data with the multicollinearity among the variables. Moreover it can deal with the situation in which the samples less than predication variates. Based on the above advantages of PLS regression, we introduce this regression to improve the crowd sensing-based security service recommendation.

**Figure 3:**
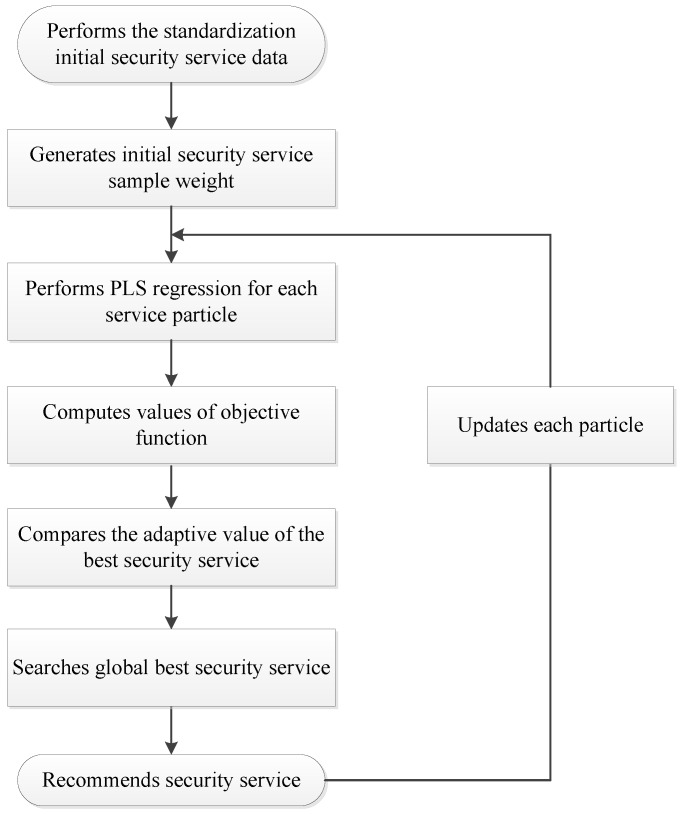
Process of the security service recommendation.

Based on the proof in [28], the PLS regression of *M^n^*, *N^n^* is the same as that of *M^nn^*, *N^nn^*. Thus the computation of sample weights between *M* and *N* can translate into the computation of the PLS regression between *M^nn^* and *N^nn^*. It is necessary to obtain service particles *p* = [*p*^1^, *p*^2^, …, *p*^3^]*^T^* and get the best predication precision, which is a global optimization problem. To resolve aforementioned problem, the objective function is set as:

(21) min  Re=∑i=1n(N⌢−Ni)2

where $\overset{⌢}{N}$ is combination predication value of *N_i_*. The process of the security service recommendation for social fog is shown in Figure 3.

**Step 1:** Standardization is performed for the initial security service data based on Equations (17) and (18).

**Step 2:** Initial security service sample weight *p* with *x*-dimension is generated, which means *x* initial particles are generated for the crowd sensing algorithm. At the same time, all parameters of the crowd sensing algorithm are initialized.

**Step 3:** *M^nn^* and *N^nn^* are computed based on *p*. For each particle, PLS regression is performed on *M^nn^* and *N^nn^*. Then the weights are obtained for each predication method.

**Step 4:** The value of objective function is computed based Equation (21), which act as the adaptive degree for each service particle.

**Step 5:** For each particle, the adaptive value is compared with the best security service it applied. If the adaptive value is better, it is set as the current best security service.

**Step 6:** For each service particle, the adaptive value is compared with the best security service of all the uses applied. If the adaptive degree is better, it is set as the current global best security service, which is recommended to the users.

**Step 7:** Each particle is updated based on Equations (15) and (16) then jump to Step 3 again.

## 5. Evaluation

### 5.1. Simulation Settings

This section presents the simulations and analysis of the performance of the proposed security service recommendation mechanism. We adapt the social relations among the fogs based on an existing dataset of social networks. The dataset of social networks can be presented, and the characteristics of the dataset can be mapped to the directions and weights. The undirected and unweighted graphs present the algorithm without the confidential relations. To simulate the social relationship among the nodes, connection attributions selected from existing social network data from Amazon are used, which is from the Stanford Network Analysis Project (SNAP) [29]. In fact, the connection attribution data of Amazon is the dimension of the parameter in our simulation for establishing a social relationship model among fog nodes. To perform the simulation and evaluation, precision rate *Prec*, recall rate *Rec*, and *F*1-measure are used as the evaluation parameters. Assume that *SeS* is the service set which satisfies the security requirements. Additionally, assume that *ReS* denotes the recalled service set of the proposed service recommendation mechanism. Furthermore, *InS* is the correct recommendation results of the proposed mechanism, which is computed by InS=SeS∩ReS. As a matter of fact, the precision rate is one of the most important dimensions in evaluating the recommendation quality of the proposed scheme. Most service recommendation schemes use the precision rate to evaluate the quality of a recommendation. The precision rate is the ratio of correct recommendation results to the recalled service set. However, the recall rate is the rate of correct recommendations to the service set which satisfies the security requirements. We added corresponding explanations to describe the precision rate. The above evaluation parameters can be computed by:

(22) $$Prec=\frac{InS}{ReS},\;ReS=\frac{InS}{SeS}$$

(23) $$F1-measure=\frac{2\cdot Prec\cdot ReS}{Prec+ReS}$$

### 5.2. Simulation Results and Analysis

In this section, simulations are done to evaluate the service recommendation delay, precision rate *Prec* and *F*1-measure. Each node in the social fog has a neighbor list, and a couple of neighbor nodes can exchange data each other directly. The number of services nodes is 10,000*d*, where *d* is an adjustment factor. To avoid the messages are transferred limitlessly in the system and waste resources, 300*z* is set as the hop count of the message forwarding, where *z* is an adjustment factor. To perform the comparison, the service recommendation method in [30] is introduced. In fact, security service recommendations are a novel topic for fog computing. There is no existing security service recommendation methodology of fog computing for doing a comparison. However, recommendation is basically a problem of and methodology for finding the best choice. Therefore, the methodology in [30] can be regarded as a basic recommendation methodology, which can also be used in service recommendation. The work in [30] proposed a top-N recommendation scheme, in which the trade-off between diversity and matching quality is formulated as a binary optimization problem, which can also be used in service recommendation. The work in [30] has an input control parameter allowing explicit tuning of this trade-off. This work is introduced for comparisons because of the importance of the control parameter in obtaining desired system performance. This scheme is introduced for comparisons because the importance of the control parameter in obtaining the desired system performance. Additionally, in the proposed scheme, particle swarm optimization (PSO) is used as a bionic evolutionary algorithm for crowd sensing in which the control parameters for recommendations are also based on the PSO algorithm. Therefore, the proposed scheme is compared with the scheme in [30]. There are some existing schemes focusing on the importance factor of a service, such as [31]. When the evaluation is done, the multi-criteria decision-making (MCDM)-based importance factor criteria in [31] is used. The simulation roadmaps of many existing works can be borrowed [32,33] Because there is instability for single tests, each test is repeated for 50 simulations. Moreover, based on the method of [34], the average value is used as a test value in the simulation. In addition, the confidence interval is set as 0.95.

**Figure 4:**
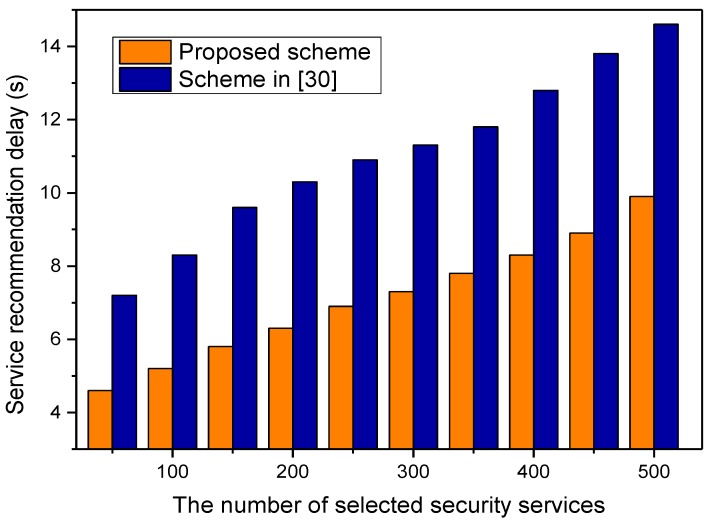
Security service recommendation delay.

**Figure 5:**
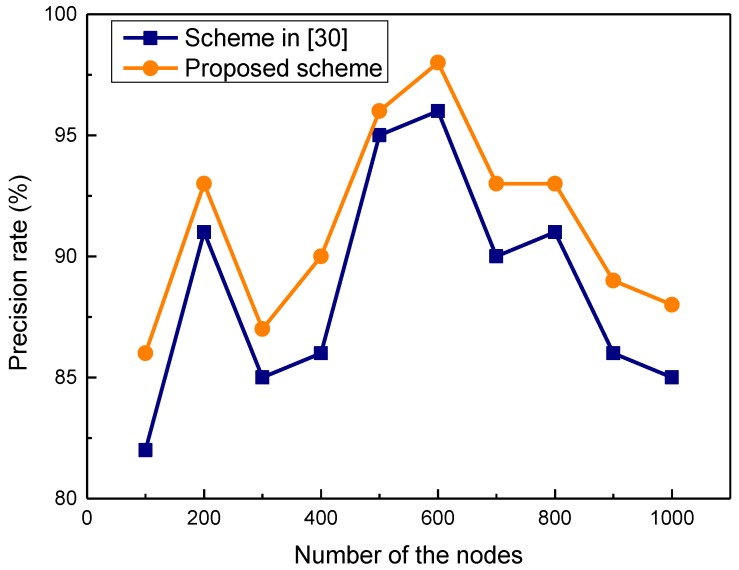
Precision comparison of security recommendation.

**Figure 6:**
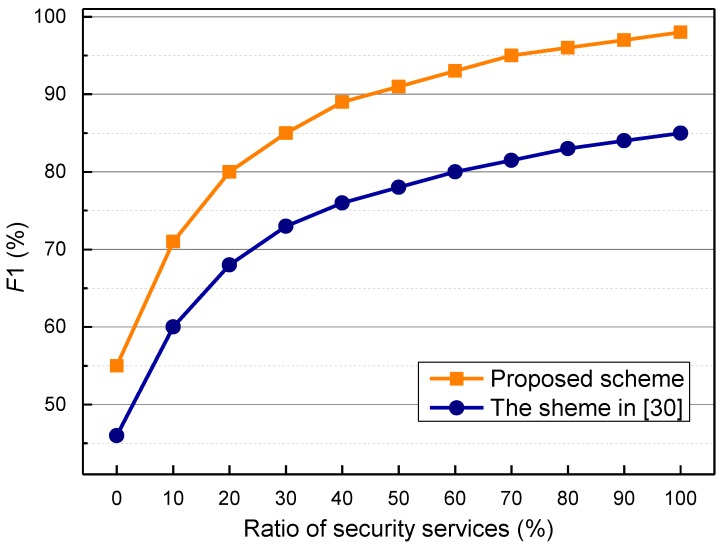
*F*1-measure of service recommendation.

We take d = 0.7 and z = 0.5 as an example of the parameter setting for the simulation. In fact, these two auxiliary adjustment factors are not the main parameters in the security services recommendation algorithm. In other words, *d* and *z* have no important impact on the security recommendation algorithm, and are just given for the running of simulations. The comparisons of security service recommendation delay are shown in Figure 4. As shown in the figure, the service recommendation delay of the proposed scheme is obviously lower than that of the scheme in [30]. In the proposed scheme, robustness and prediction capabilities can be obtained based on PSO and chance discovery; thus, the recommendation speed of the proposed scheme is faster. In Figure 5, it is obvious that the precision rate of the proposed scheme is higher than that of [30]. This is because the proposed security mechanism is based on chance discovery and crowd sensing. The recommendation faults are caused by the dynamics of the nodes and communications, which make the recommendations unavailable or have inconsistent descriptions. Next, evaluations and comparison of the *F*1-measure are performed. The simulation results of the *F*1-measure dependent on the security service ratio are shown in Figure 6. When the ratio of security service is lower than 20%, the value of the *F*1-measure is obviously low. If the ratio of security service is higher than 20%, the value of the *F*1-measure increases obviously and is near its peak when the *F*1-measure is more than 98%. The *F*1-measure of the proposed scheme is higher obviously than that of [30].

## 6. Conclusions

It is a new trend to combine social network with existing communication networks, cloud systems, etc. In fact, the social organization of networks can enable more flexible, secure, and collaborative performance for networking and computing, which make the social network a potential architecture for fog computing systems. It is very necessary to adapt fog computing based on social architecture. To address this, we designed a social networking advanced architecture for fog computing. Moreover, to satisfy the security requirements of the proposed social fog computing, a crowd sensing-enabling security service recommendation method was proposed including security service assessment, discovery, and recommendation. The security assessment on services is performed through introducing security factors, including exploitability, credibility, severity, confidentiality, integrality, availability, and importance weight. Furthermore, a KeyGraph-based chance discovery and PSO-based crowd sensing are adapted to realize the security service recommendation. The simulation results also demonstrated the efficiency of the proposed security service recommendation scheme. Based on the proposed security service recommendation scheme, it will become more convenient for fog users to share their own services, resources, and data via social networks in a secure manner. In addition, when the computing technologies develop from cloud to fog, the implementation schemes (e.g., live virtual machine migration [35]) is important issues. Therefore, efficient virtual machine migration scheme for social fog is our future work.
